# Donor age negatively impacts adipose tissue-derived mesenchymal stem cell expansion and differentiation

**DOI:** 10.1186/1479-5876-12-8

**Published:** 2014-01-07

**Authors:** Mahmood S Choudhery, Michael Badowski, Angela Muise, John Pierce, David T Harris

**Affiliations:** 1 Advanced Centre of Research in Biomedical Sciences, King Edward Medical University, Lahore, Pakistan; 2 Department of Immunobiology, College of Medicine, The University of Arizona, PO Box 245221, 85724, Tucson, AZ, USA; 3 Aesthetic Surgery of Tucson, Tucson, AZ, USA

**Keywords:** Adipose tissue, Mesenchymal stem cells, Donor age, Regenerative potential, Growth kinetics, In vitro differentiation potential

## Abstract

**Background:**

Human adipose tissue is an ideal autologous source of mesenchymal stem cells (MSCs) for various regenerative medicine and tissue engineering strategies. Aged patients are one of the primary target populations for many promising applications. It has long been known that advanced age is negatively correlated with an organism’s reparative and regenerative potential, but little and conflicting information is available about the effects of age on the quality of human adipose tissue derived MSCs (hAT-MSCs).

**Methods:**

To study the influence of age, the expansion and in vitro differentiation potential of hAT-MSCs from young (<30 years), adult (35-50 years) and aged (>60 years) individuals were investigated. MSCs were characterized for expression of the genes p16^INK4a^ and p21 along with measurements of population doublings (PD), superoxide dismutase (SOD) activity, cellular senescence and differentiation potential.

**Results:**

Aged MSCs displayed senescent features when compared with cells isolated from young donors, concomitant with reduced viability and proliferation. These features were also associated with significantly reduced differentiation potential in aged MSCs compared to young MSCs.

**Conclusions:**

In conclusion, advancing age negatively impacts stem cell function and such age related alterations may be detrimental for successful stem cell therapies.

## Background

The average human life expectancy has significantly increased due to advances in medical research and improvements in general life style. Unfortunately however, human aging is associated with many clinical disorders and an inability of the body to maintain tissue turnover and homeostasis. As a result the number of elderly medical patients have also significantly increased, making them a major target population that could potentially benefit from cell based therapies. As autologous cell sources are preferred for economical and logistical reasons (along with fewer potential side-effects), the effect of donor age on regenerative potential should be determined before clinical use. In recent years, many studies have demonstrated the clinical potential of mesenchymal stem cells (MSCs), both in vivo and in vitro [1,2]. However, using MSC collected from the elderly who are most likely to benefit from this technology raises some practical concerns.

MSCs possess a multitude of potential applications in regenerative medicine, being able to proliferate and differentiate in vitro into multiple lineages [3-5]. Low immunoreactivity and high immunosuppressive properties make MSCs a suitable stem cell source for therapy [6,7]. In various animal models it has been shown that MSCs can be used to successfully treat cardiovascular [2,8], neurological [9] and musculoskeletal disorders [10] either by differentiation into competent cardiomyocytes, neuron-like cells and chondrocytes, respectively; or by a paracrine effect via the secretion of growth, anti-apoptotic and anti-inflammatory factors. To date, bone marrow is the best characterized source of MSCs and most clinical data is based on bone marrow studies. However, there are limitations to the use of bone marrow-derived MSCs (BM-MSCs), e.g. painful acquisition process, use of extensive anesthesia, and low cell yield. Murine BM-MSCs have been shown to exhibit a decline in MSC numbers, proliferation, angiogenic and wound healing properties, and differentiation, along with enhanced apoptotic and senescent traits [2,11,12] with advancing donor age. Recently, other MSC sources have gained clinical interest for use in regenerative medicine; and adipose tissue represents one of these sources with a broad spectrum of benefits. hAT-MSCs possess morphological, phenotypic and functional characteristics similar to BM-MSC [13], are stable over long term culture, expand efficiently in vitro and possess multi-lineage differentiation potential [5,14]. Human adipose tissue represents a more practical autologous source of MSCs for various tissue engineering strategies. However, the effectiveness of these cells when obtained from and utilized in elderly patients must be considered when contemplating cell-based therapies.

Cell-based therapies will be significantly influenced by the expansion and differentiation potential of any cells to be used which in turn may be influenced by donor age. In the present report, we thus sought to study the growth characteristics and in vitro regenerative potential of hAT-MSCs obtained from donors of various age groups (young, adult and aged) in combination with gene expression profiles, superoxide dismutase (SOD) activity and senescence levels. Morphologically and immunophenotypically cells obtained from all donors were similar regardless of donor age. However, we observed significant decreases in MSC number, frequency and population doublings concomitant with an increase in senescence levels with increasing donor age. Our qualitative and quantitative observations indicated that although adipogenic (and possibly neurogenic) potential was maintained during advancing age, the osteogenic and chondrogenic abilities were negatively affected by donor age.

## Methods

### Collection and processing of adipose tissue-derived mesenchymal stem cells

**Figure 1:**
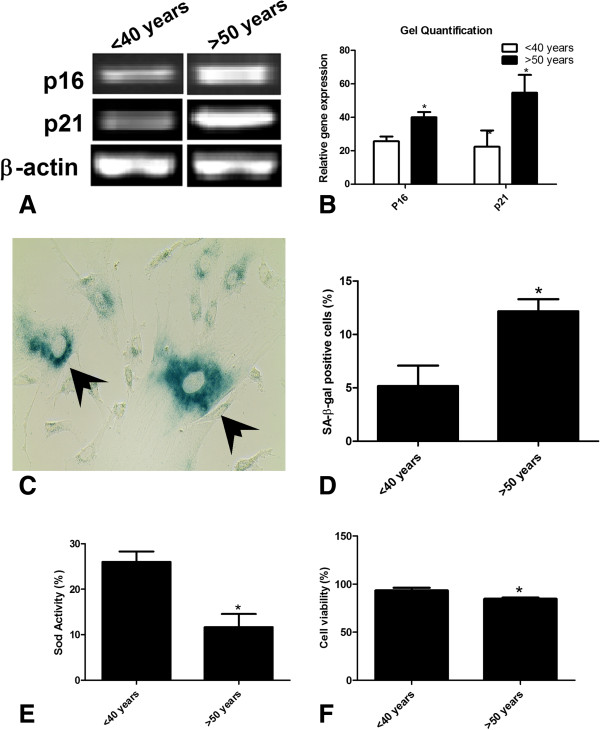
**Comparison of age related parameters in AT-MSCs isolated from young and aged donors. (A, B)** Gene profiling of AT-MSCs indicate that gene expression of p16 and p21 is higher is AT-MSCs isolated from aged as compared to young donors. Data is shown as fold-induction as compared to untreated control cells. **(C, D)** Similarly, aged cultures show senescent features as determined by SA-β-gal staining compared to young cultures (data shown as percent positive cells). Concomitant with these features young AT-MSCs have higher level of SOD (**E**; data shown as absorbance values) and viability after stress (**F**; data shown as percent viable cells). Results are expressed as Mean ± Standard deviation. *P < 0.01 for young AT-MSCs versus aged AT-MSCs. AT-MSCs: adipose tissue derived mesenchymal stem cells, SA-β-gal: senescence associated beta galactosidase.

Consent was obtained from all donors before the liposuction procedure. All protocols were approved by the local Institutional Review Board (IRB). Human adipose tissue was obtained from liposuction procedures under local anesthesia. All adipose tissue samples were processed under the same conditions. Initial experiments (see Figure 1 and Additional file 1: Figure S1) were performed using subjects divided into <40 years of age (N = 5; 2 males and 3 females) or > 50 years of age (N = 6; 1 male and 5 females) to examine if there were indeed any effects of donor age on AT-MSC. In subsequent experiments additional donors were recruited and divided into 3 age groups: group 1 (<30 years; n = 8; mean age 25.5 ± 1.6 years; 4 males and 4 females), group 2 (35-55 years; n = 10, mean age 46.4 ± 2.1 years; 3 males and 7 females) and group 3 (> 60 years; N = 11; mean age 66.0 ± 1.4; 2 males and 9 females).

The raw lipoaspirate was processed using a previously described method [5]. Cells from adipose tissue samples were isolated by enzymatic digestion. Briefly, 2 mL of tissue slurry was placed in a 50-mL Falcon tube and washed vigorously five times with 5 mL of phosphate-buffered saline (PBS). Cells in the wash fraction were retained. The fatty tissue was treated with an equal volume of 0.2% collagenase type IV (Sigma) at 37°C for 15 min. Complete medium (Minimal Essential Medium; Thermo Scientific, USA), 20 mL, supplemented with 10% fetal bovine serum (FBS; Hyclone) and 1% each of non-essential amino acids, sodium pyruvate, glutamine and streptomycin/penicillin solution was added in the digested tissue to neutralize collagenase, passed through a 40-μm filter and centrifuged at 150 *g* for 10 min. The cells from both the wash fraction and the digested fraction were suspended in complete medium and counted using trypan blue and Turk’s stains. Cells were plated in 25 cm^2^ culture flasks and maintained at 37°C/5% CO_2_ in expansion medium with humidity. MSCs adhered to the culture flasks whereas other cells were depleted by replacing the spent medium with fresh medium. The medium was changed twice a week thereafter. To prevent spontaneous differentiation, cells were maintained at sub-confluent levels (70-80%) and were harvested with 0.05% trypsin-EDTA for use in subsequent experiments.

### Phenotypic characterization by flow cytometry

Cultured cells (passage 1) were trypsinized and stained with a panel of antibodies for fluorescence-activated cell sorting (FACS) analysis. Approximately, 1 × 10^5^ cells were re-suspended in phosphate buffered saline (PBS) and incubated with IgG block for 5 minutes to block non-specific binding. The following antibodies were used: AF-700 conjugated CD3 (BD BioSciences, USA), PE conjugated CD14 (BD, Immunocytometry, USA), APC conjugated CD19 (BD BioSciences, USA), PE conjugated CD34 (BD, BioSciences, USA), APC conjugated CD44 (BD, Pharmingen, USA), FITC conjugated CD45 (BD Pharmingen, USA), PE conjugated CD73 (BD Pharmingen, USA), AF-700 conjugated CD90 (Biolegend, USA) and APC conjugated CD105 (Biolegend, USA). Cells were stained for 30 minutes at 4°C with the antibodies. After washing, samples were analyzed on a LSR II flow cytometer (BD, USA) and at least 10,000 events were acquired for each population. Data acquisition and analysis were performed using FACS DIVA software (BD Biosciences, USA). Unstained cells were used to establish flow cytometer settings. Debris and cells/particles with auto-fluorescence were removed by using a threshold on the forward scatter.

### Cell proliferation assays

#### Assay for colony forming unit (CFU-assay)

CFU-assays were performed to determine MSC frequency. The processed lipoaspirates after collagenase digestion were plated in 25 cm^2^ culture flasks in limiting dilutions (10^5^, 5 × 10^4^, 10^4^, etc.) to verify the ability to form colonies. Cultures were maintained for 14 days at 37°C/5% CO_2_ in expansion medium. At day 14, medium was removed and resultant colonies were washed twice with PBS, fixed with absolute methanol and stained with 0.1% crystal violet for 60 minutes at room temperature [5]. The flasks were washed with water and colonies with more than 30 cells were counted under a microscope by two independent observers.

#### Cumulative growth index

MSCs were serially passaged for cumulative population doubling analysis as described [2]. The first confluent cultures were designated as passage 0 (P0) and were dissociated with trypsin/EDTA, counted by hemacytometer and re-plated at a 1:10 dilution. The cell number was recorded for each passage until the cells stop dividing. The average cell number was expressed with respect to time in culture to obtain a growth curve. The population doublings (PDs) and doubling time (DT) were calculated using the following equations [2],

$$\mathrm{PDs}=\mathrm{Log}\;\left(N/N_{0}\right)\times 3.31$$

DT=CT/PDs

Where, PDs represent population doublings, N is the final number of cells, N_o_ is the initial number of cells seeded DT is doubling time and CT is the time in culture.

#### In vitro differentiation assays

MSCs from young, adult and aged groups were analyzed for the potential to differentiate into adipose, bone, cartilage and neuron-like cells. MSCs were induced to differentiate between passages 2-3, as described below.

#### Adipogenic differentiation

AT-MSCs were seeded in triplicate in 12 well plates at a final cell density of 5,000 cells per cm^2^ in complete expansion medium. 24-48 hours later, which was designated as day 0, differentiation was initiated using adipogenic induction medium (ThermoScientific, USA), as per manufacturer’s instructions. The medium was changed every 3-4 days thereafter and experiments were terminated after 3 weeks. AT-MSCs at the same cell density were maintained in expansion medium to serve as controls.

#### Oil red O staining

Adipogenesis was confirmed three weeks after induction by oil Red O staining to visualize accumulated cytoplasmic lipid rich vacuoles [5,14] according to the manufacturer’s instructions (IHC World, USA). Briefly, the differentiated MSCs were fixed with 4% paraformaldehyde (PFA), washed with pre-stain solution (99% isopropanol) and incubated with oil red O solution for 30 minutes at 60°C. Oil red O staining was followed by washing with 60% isopropanol and then several changes of distilled water. Cells were counterstained with haematoxylin solution for 1 minute and visualized under phase contrast microscopy.

#### In vitro osteogenic differentiation

For osteogenic differentiation 50,000 MSCs per well were seeded in 6 well plates in triplicate in expansion medium. After 24 hours (at 90% confluency) osteogenic differentiation was promoted by treating MSC cultures with osteogenic induction medium (ThermoScientific, USA) for 3 weeks while MSCs maintained in expansion medium for 3 weeks were used as controls.

#### Von Kossa staining

Osteogenic potential was confirmed by the von Kossa’s method [15] of extracellular matrix calcification detection. Von Kossa’s staining was performed by silver nitrate treatment using a commercially available kit (IHC World, USA). Briefly, PFA (4%) fixed cultures were treated with silver nitrate for 60 minutes at room temperature under ultraviolet (UV) light, followed by treatment with sodium thiosulphate for 5 minutes. The cells were counter-stained with nuclear fast red and then photographed using phase contrast microscopy. Extracellular matrix calcification was carried out by detection of the presence of black extracellular deposits.

#### Chondrogenic differentiation

Chondrogenesis was induced in micromass pellet cultures as described [5,16]. Micromass-pellet cultures were prepared from 2.5 × 10^5^ MSCs in 15 ml conical tubes that were centrifuged at 750 rpm for 10 minutes in complete expansion medium. Cell pellets were incubated with the chondrogenic induction medium (ThermoScientific, USA) after 24-48 hours for 3 weeks. Half of the medium was replaced with fresh medium twice a week.

#### Alcian blue staining

Each micromass pellet was parafinized after dehydration and cut into thin sections (4-5 um). The sections were analyzed for chondrogenic differentiation assay with a commercially available Alcian blue kit (IHC World, USA). Sections were fixed with 4% PFA and washed with distilled water followed by treatment with Alcian blue for 20 minutes at room temperature. The stained sections were visualized under phase contrast microscopy and images were captured.

#### Differentiation of MSCs into neuron-like cells

2.5 × 10^3^ MSCs at passage 2 were propagated in six well plates in complete growth medium, followed by treatment with neuronal induction medium (ThermoScientific, USA) as per manufacturer’s instructions. After 24-48 hours, the differentiated MSCs were stained with H&E (Hematoxyline and Eosin). Briefly, the medium was discarded and the cells were washed with PBS, fixed with methanol and stained with H&E solution for 30-60 seconds, and photographed using phase contrast microscopy. Cells having a neuron-like morphology were counted in each culture.

#### Quantification of differentiation

The total number of oil red O positive MSCs were counted in triplicate in at least 10 non-overlapping high density fields. The mean differentiation level (%) was expressed as total number of oil red O positive cells divided by total number of cells, and then multiplied by 100. Additionally, oil red O uptake was quantified colorimetrically using a previously published method [17]. Briefly, oil red O was extracted with isopropanol containing 4% nonidet P-40 detergent overnight at room temperature and optical density was then measured at 520 nm [5,17]. All analyses were carried out in triplicate.

For the quantification of mineralized matrix deposition, imageJ software (http://rsbweb.nih.gov/ij/) was used which measures the amount of cellular staining (black) in a given field of view. Percentage positive area was calculated by dividing the positively stained area divided by the total area, multiplied by 100. All analyses were carried out in triplicate.

Alcian blue uptake was analyzed using a colorimetric assay as described [18]. Briefly, after 21 days, the micromass cultures were fixed with methanol and the whole mount stained with Alcian blue. Alcian blue was extracted with 6 M guanidine HCl and absorbance was read at 620 nm.

#### Senescence-associated β-galactosidase Staining (SA-β-gal)

To detect cellular senescence the SA-β-gal staining kit was used (Cell Signalling, USA). Briefly, 5 × 10^3^ cells were seeded in 12 well plates incubated with freshly prepared β-gal-staining solution for 60 minutes at 37°C in the absence of CO_2_. MSCs were washed with water and the blue color (i.e., senescent cells) was observed under microscopy. Phase contrast images were taken and the percentage SA-β-gal positive cells were calculated by dividing blue stained cells by the total number of cells, multiplied by 100.

#### Superoxide dismutase (SOD) activity

Age-related differences in SOD activity were determined using a commercially available colorimetric assay kit (Abcam, USA) according to the protocol provided by the manufacturer. Briefly, protein was isolated using a lysis buffer and SOD activity was measured using 10 ug of the total protein extract. Absorbance values were measured by using a Spectra max PLUS 384 (Molecular Devices, USA) at 450 nm.

#### Immunocytochemistry

After culture in neuronal differentiation medium, differentiated cells were washed with PBS and incubated with the rabbit-anti-human nestin (US Biological, USA) overnight at 4°C. The cells were then washed with PBS and incubated with PE-conjugated goat-anti-rabbit (Santa Cruz, USA) antibody for 60 minutes at 37°C. Cells were mounted using vecta-sheild mounting medium (Vector Laboratories, USA) containing DAPI (4’,6-diamidino-2-phenylindole) to stain nuclei and observed using fluorescence microscopy (Zeiss, USA).

#### Total RNA extraction

Gene expression was assayed at the mRNA level. Total cellular RNA was extracted using TRIzol reagent (Invitrogen, USA) and an Rneasy Mini Kit (Qiagen, USA). All procedures were carried out according to the protocol recommended by manufacturers. RNA concentration was determined using a ND-1000 spectrophotometer (NanoDrop Technologies). cDNA synthesis was performed by using 1 ug of total RNA in a 20 ul reaction mixture containing 1 ul of 10 uM oligodt primer and 1 ul of reverse transcriptase enzyme (RT-enzyme) with the SuperScript III First Strand synthesis system (Invitrogen, USA). The manufacturer’s instructions were followed.

#### Quantitative RT-PCR

Real time PCR was performed using iTaq SYBR Green supermix with ROX (Bio-Rad, USA) in an ABI PRISM 7300 sequence detection system. The final reaction contained template cDNA, iTaq SYBR Green and gene specific primers (see Additional file 2: Table S1). The following PCR conditions were used: 50°C for 2 minutes and 95°C for 10 minutes, followed by 40 cycles for 30 sec at 95°C, 45 sec at 60°C and 72°C for 30 sec. Beta actin was used as an internal control. The CT (cycle threshold) values of beta actin and other specific genes were acquired after polymerase chain reaction. The normalized fold expression was obtained using the 2^-ΔΔCT^ method. The results were expressed as the normalized fold expression for each gene as compared to untreated (i.e., un-induced) control cells. In order to minimize PCR reaction variations, all samples were transcribed simultaneously.

#### Cell viability

To determine the level of cell viability in response to incubation in H_2_O_2_, the trypan blue exclusion assay was used. Sub-confluent cultures of MSCs were incubated with 200 um H_2_O_2_ for 90 minutes. Cells were trypsinizedand counted in a hemacytometer. The number of viable cells was calculated by dividing the number of trypan blue-negative cells by the total number of cells examined, multiplied by 100.

### Statistical analysis

Experimental data was analyzed using Graphpad Prism 5 Software. One-way ANOVA was used when three or more groups within one variable were compared. To analyze two groups the unpaired-t-test was used. The data are expressed as mean ± standard deviation. Values of P <0.05 were considered significant.

## Results

### AT-MSC morphology and phenotype is independent of donor age

MSCs from each age group exhibited identical morphological and phenotypic characteristics, as well as similar plastic adherent growth and fibroblastic morphology, independent of donor age. Similarly, MSCs from all age groups displayed comparable levels of expression of CD3, CD14, CD19, CD34, CD44, CD45, CD73, CD90 and CD105 (Additional file 1: Figure S1A). Regardless of donor age MSCs were strongly positive for MSC markers (CD44, CD73, CD90, CD105) while lacking expression of hematopoietic markers (CD3, CD14, CD19, CD34, CD45) (Additional file 1: Figure S1A); in agreement with previously published reports [19,20]. Additional file 1: Figure S1B shows the relative percentage expression for each marker used in the experiment.

### Cellular senescence increases in elderly AT-MSC

Senescence was evaluated in two ways: by measuring mRNA expression of the p16 and p21 genes and, by detection of SA-β-gal expression [2,12]. Initially, two groups of donors (<40 years old and >50 years old donors) were examined. The mRNA levels of the p16 and p21 genes (Figure 1A), which are associated with senescence, were analyzed by PCR [21]. The expression of both genes was significantly higher in the aged group (>50 years) than in the young group (<40 years) as shown in Figure 1B (p < 0.05). SA-β-gal activity is the most commonly used biomarker for the identification of senescent cells [22]. Our results showed that AT-MSC donor age was also associated with an increase in the expression of SA-β-gal activity (Figure 1C-D), (5.2% ± 1.9% vs. 12.2% ± 1.1% SA-β-gal positive cells; p < 0.05).

### Superoxide dismutase (SOD) activity

Reactive oxygen species (ROS) are hypothesized to be involved in the aging process; therefore, age-related variations in SOD activity could substantially influence the aging process. SOD activity was determined in MSCs isolated from young (<40 years) and aged (>50 years) individuals. SOD is an antioxidant enzyme that catalyzes the conversion of superoxide radical anions (O^2-^) to hydrogen peroxide, which is then catalyzed to O_2_ and H_2_O by glutathione peroxidase and catalase. SOD activity was significantly higher in young MSCs as compared to aged cells, indicating superior antioxidant defense mechanisms in young MSCs (Figure 1E), (26.0 ± 2.3 vs. 11.7 ± 2.9; p < 0.05).

### Effect of donor age on cell viability after stress

The success of cell based therapy is largely dependent on the survival of transplanted cells into an ischemic environment. Therefore, we determined the viability of the AT-MSC cells after hypoxic insults with H_2_O_2_. H_2_O_2_ treatment resulted in significant levels of death in aged MSC cultures as compared to younger MSC cultures. The results showed a significantly higher percentage of viable MSCs isolated from young compared with aged donors (Figure 1F) (93.3% ± 2.8% vs. 84.7% ± 1.2% enzymatic activity; p < 0.05).

### Effect of donor age on cell yield, viability and MSC frequency

**Figure 2:**
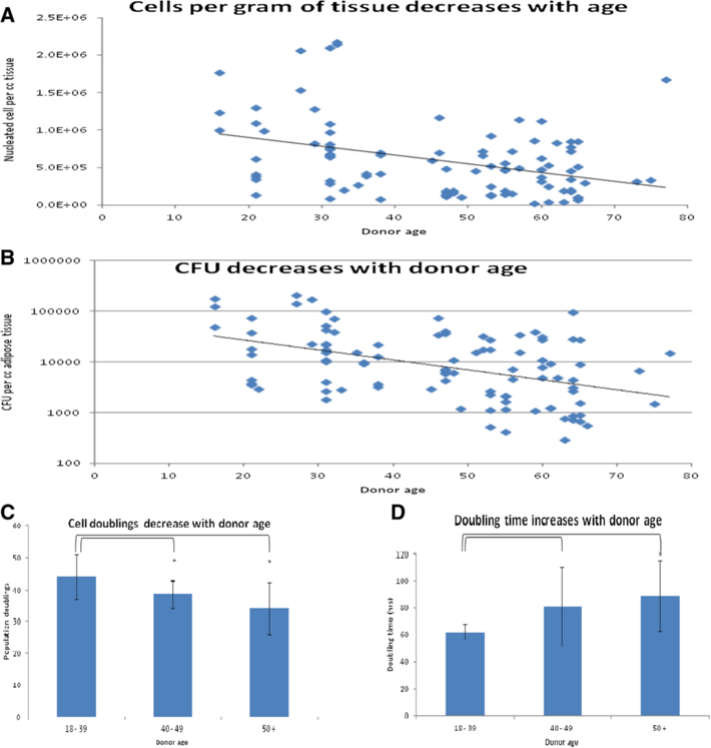
**Yield and growth characteristics of MSCs.** Cells per gram of adipose tissue decreases with increased age of the donors **(A)**. CFU assay was performed to enumerate number of cells in SVF that can form colonies. Number of CFUs deceases with age of the donor **(B)**. Proliferative potential is associated with donor age as indicated by number and time for population doublings. Number of population doublings of MSCs decreases **(C)** while time per population doubling increases **(D)** with age of the donor. Results are expressed as mean ± standard deviation. MSCs: mesenchymal stem cells, CFU, colony forming unit assay. *P < 0.01 for young AT-MSCs versus aged AT-MSCs.

**Figure 3:**
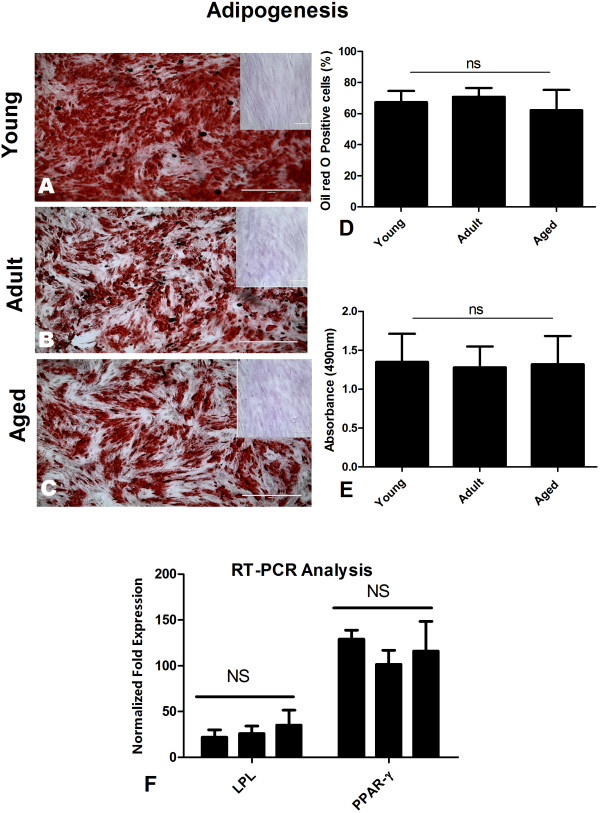
**Adipogenic potential of AT-MSCs is independent of donor age.** Adipogenic differentiation was carried out for AT-MSCs isolated from young, adult and aged individuals. Insets show MSCs cultured in normal expansion medium. Adipogenic experiments were terminated after 21 days and oil red O was used to stain for lipid rich vacuoles, as shown (Figure 3A-C). The percentage of cells that stained positive for oil red O was determined, followed by quantification of oil red O uptake. Adipogenic differentiated MSCs isolated from young **(A)**, adult **(B)** and aged **(C)** donors stained positive for oil red O. Differentiation levels varied between groups but non-significantly as indicated by counting oil red O positive cells **(D)** and colorimetrically by evaluating oil red O uptake **(E)**. Adipogenic differentiation was further confirmed through real time RT-PCR and a non-significant difference was found when different age groups were compared **(F)**. Results are expressed as mean ± standard deviation.

The number of nucleated cells in the stromal vascular fraction (SVF) of the adipose tissue samples from each donor was enumerated with Turks dye. The results indicated that adipose tissue harvested from aged donors produced the lowest number of total nucleated cells. Cell viability of the SVF, as determined by Trypan blue exclusion, was equivalent regardless of donor age. Figure 2A shows that the cells per gram of adipose tissue decreases with donor age. We next analyzed whether MSC frequency in the SVF was affected by donor age. Colony forming unit (CFU-F) assays were performed in this regard. MSCs from all groups were able to form colonies; however, an age-related decrease in MSC frequency was observed (Figure 3B). The number of MSC colonies formed was found to be inversely proportional to donor age. Overall, cells from young and adult cultures produced larger colonies containing more cells while AT-MSC from aged donors produced smaller colonies, although these results did not reach statistical significance (data not shown). However, the trend lines in panel (A) and (B) were significant indicating the total cells/gm of tissue and total CFUs/cc of tissue decreased with increasing donor age.

### Effect of donor age on AT-MSC growth

The effect of donor age on AT-MSC growth characteristics and maximum population doublings was calculated. Cell numbers recorded at each passage were used to obtain a growth curve that showed an exponential growth phase after which cell proliferation declined until the cells stopped dividing. There was a significant difference (p < 0.05 where indicated) in the number of total population doublings (Figure 2C, 44.1 ± 7.1 vs. 38.5 ± 4.3 vs. 34.3 ± 8.1 doublings for young, adult and aged donors, respectively), with doubling times increasing with increased donor age (Figure 2D, 62.0 ± 5.9, 80.9 ± 29.6 and 89.1 ± 26.6 hours for young, adult and aged donors, respectively).

### Assay for multi-lineage differentiation potential

Previously, we and others have shown that AT-MSCs have the potential to differentiate into multiple lineages in vitro [5,14]. Thus, MSC from each age group were induced to differentiate into adipogenic, osteogenic, chondrogenic and neurogenic lineages.

### Adipogenic differentiation potential of AT-MSCs is independent of donor age

Under adipogenic conditions MSCs showed significant morphological changes as early as 7 days in culture. The typical spindle-like shape was progressively lost and cells exhibited a larger, flatter shape full of lipid vacuoles. No related morphological differences were observed between age groups (Figure 3A-C). Insets show MSCs cultured in normal expansion medium. Adipogenic experiments were terminated after 21 days and oil red O was used to stain for lipid rich vacuoles [5,20], as shown (Figure 3A-C). The percentage of cells that stained positive for oil red O was determined. The percentage of cells positive for oil red O expression [20] was higher in the young donors (Figure 3A) than in the other groups (Figure 3B-C), but this difference was not significant (Figure 3D) (67.4% ± 7.1% in young vs. 70.8% ± 5.6% and 62.2% ± 13.0% positive, respectively in adult and aged). Quantification of oil red O uptake indicated a statistically non-significant difference among MSCs obtained from young, adult and aged individuals (Figure 3E). Histochemistry findings were confirmed by real time RT-PCR analysis. The expression of the adipogenesis-specific genes, peroxisome proliferator-activated-receptor-gamma (PPAR-ү) and lipoprotein lipase (LPL), was analyzed [17,20]. The expression of both adipogenic specific genes (LPL: 22.0 ± 8.1 (young), 26.0 ± 8.3 (adult), 35.3 ± 16.2 (aged) fold-expression, and PPAR-ү: 129.3 ± 9.5 (young), 101.7 ± 15.3 (adult), 116.0 ± 32.6 (aged) fold-expression) was significantly higher in induced MSCs as compared to control MSCs. However, there was no significant difference among the various age groups (Figure 3F). Overall our findings indicated that the adipogenic potential of AT-MSCs was independent of donor age and thus it appeared that AT-MSCs could potentially be used for tissue engineering applications that require adipocyte production without concern for age of the donor.

### AT-MSC osteogenic potential declines with donor age

**Figure 4:**
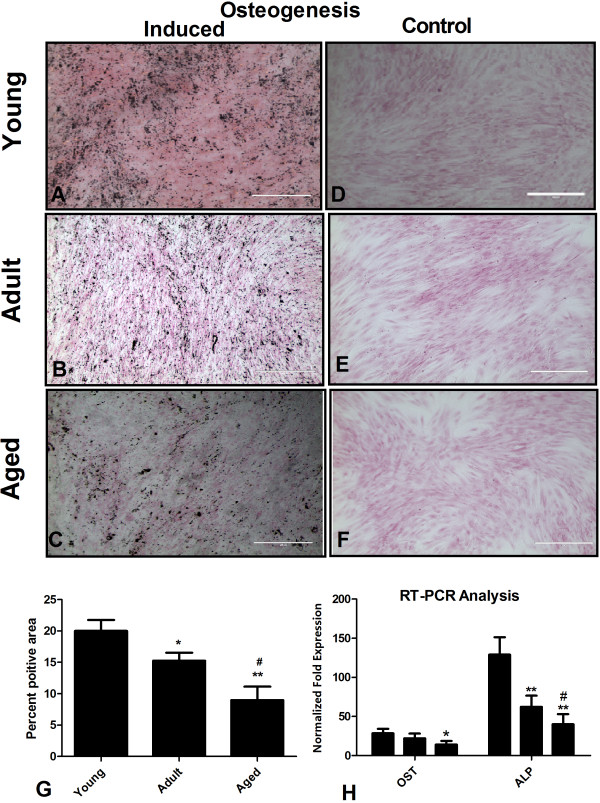
**Effect of donor age on osteogenic potential of AT-MSCs.** Osteogenic induction was assessed by von Kossa staining. **(A-C)** Representative figures showing matrix mineralization in induced cultures of young, adult and aged, respectively. **(D-F)** Control AT-MSCs did not stain positive with von Kossa staining. Possible age related differences in osteogenic potential were measured using ImageJ software. AT-MSCs isolated from young individuals revealed more matrix mineralization than adult and aged groups **(G)**. Similar, results were obtained when gene expression of OST and ALP was analyzed through quantitative RT-PCR **(H)**. Results are expressed as mean ± standard deviation. *P < 0.05, **P < 0.01, ***P < 0.001 for young AT-MSCs versus aged AT-MSCs, ^#^P for adult versus aged AT-MSCs. OST: osteocalcin, ALP: Alkaline phosphatase.

AT-MSCs isolated from each age group were induced to differentiate into osteoblasts. The cells proliferated rapidly in a tightly packed monolayer culture to form aggregates with calcium deposition. After 3 weeks, Von Kossa staining [15] revealed a positive extracellular matrix formation in the induced AT-MSCs (Figure 4A-C). AT-MSCs obtained from younger (Figure 4A) donors produced more matrix than AT-MSCs obtained from both adult (Figure 4B) and aged (Figure 4C) donors. Cells in the control group did not show such changes (Figure 4D-F). Comparative quantification of Von Kossa staining by ImageJ software is shown for all age groups (Figure 4G). Significant age-related differences among young (20.0% ± 1.7% positive cells), adult (15.2% ± 1.3% positive cells) and aged (8.9% ± 2.2% positive cells) were observed in this regard. Osteogenic induction was further evaluated by real time RT-PCR analysis of lineage-specific expression of two osteogenic genes, alkaline phosphatase and osteocalcin [23]. When comparing the effect of donor age on osteogenic potential, we observed a higher expression of the osteogenic specific genes in young AT-MSC compared to the other age groups (Figure 4H), (Osteocalcin: 28.3 ± 5.9 (young), 22.0 ± 6.1 (adult), 11.7 ± 1.4 (aged) fold-expression, and Alkaline phosphatase: 129.0 ± 14.6 (young), 63.7 ± 3.2 (adult), 36.3 ± 5.2 (aged) fold-expression).

### Chondrogenic potential of AT-MSCs declines with age

**Figure 5:**
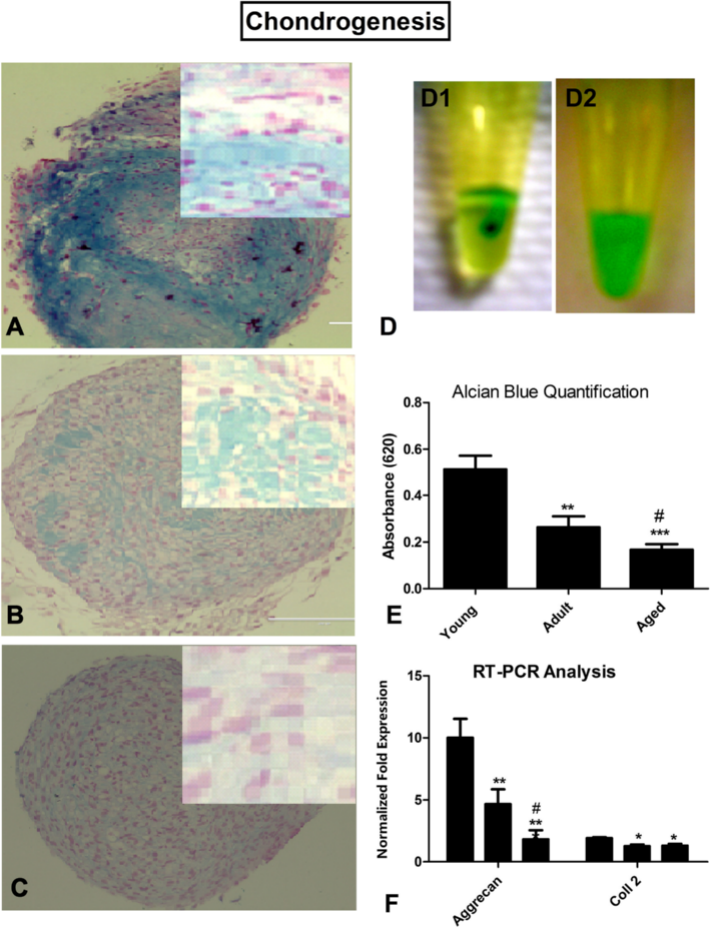
**Chondrogenic potential of AT-MSCs is compromised by donor age.** Differentiation of AT-MSCs was induced chondrogenic induction medium for 21 days in micromass pellet culture. Alcian blue staining was performed for induced AT-MSCs isolated from **(A)** young, **(B)** adult and **(C)** aged individuals. At higher microscopic magnification (shown in insets) the cartilage like tissue appeared to be composed of rounded cells, surrounded by lacunae lying in a proteoglycan rich extracellular matrix. Chondrogenic in vitro potential was quantified by a colorimetric assay in which Alcian blue uptake (blue color) was extracted with 6 M guanidine HCl and absorbance was read at 620 nm. **(D1)** showing micromass 30 minutes after incubation and **(D2)** after 120 minutes. **(E)** absorbance values were significantly higher for young and adult AT-MSCs compared to aged. **(F)** Quantitative RT-PCR was performed for mRNA expression of aggrecan and collagen type 2. Age related decline in mRNA level was observed for both genes. Results are expressed as mean ± standard deviation. *P < 0.05, **P < 0.01, ***P < 0.001 for young AT-MSCs versus aged AT-MSCs, ^#^P for adult versus aged AT-MSCs.

Chondrogenic differentiation of AT-MSCs was performed in micromass pellet cultures. Thin sections of the pellets were stained with Alcian blue (Figure 5A-C) to detect sulfated proteoglycans in the cartilage matrix [24]. Uptake of Alcian blue staining was quantified colorimetrically (Figure 5D-E) and quantitatively (Figure 5F). Differences were observed when comparing AT-MSCs obtained from the various age groups (0.51 ± 0.06 vs. 0.26 ± 0.04 vs. 0.17 ± 0.02 OD units). At higher microscopic magnification (insets) the cartilage-like tissue appeared to be composed of rounded cells, surrounded by lacunae and lying in a proteoglycan rich extracellular matrix [24,25]. Significant age-related differences were observed in the chondrogenic differentiation potential of AT-MSCs isolated from various age groups when real time RT-PCR analysis (Figure 5F) was performed using the lineage specific genes, aggrecan and collagen type 2 [26,27] (Aggrecan: 10.0 ± 1.5 (young), 4.3 ± 0.2 (adult), 1.8 ± 0.4 (aged) fold-expression, and Collagen type 2: 1.9 ± 0.1 (young), 1.3 ± 0.1 (adult), 1.3 ± 0.2 (aged) fold-expression). These latter two findings indicated that donor age negatively regulated AT-MSC differentiation into cartilage.

### AT-MSCs undergo a neuronal-like differentiation in vitro independent of donor age

**Figure 6:**
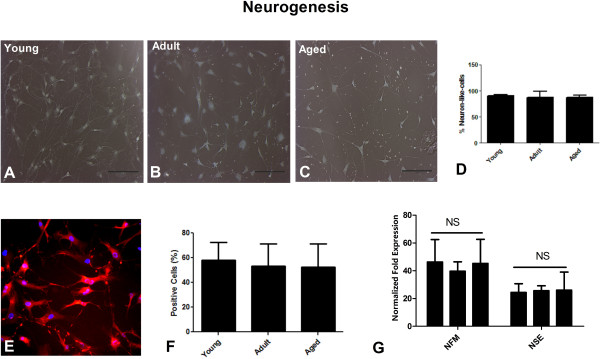
**The neuron-cell-like morphology of AT-MSCs isolated from young, adult and aged donors. (A-C)** Neuron-like-morphology was observed in MSCs isolated from young, adult and aged individuals, respectively. **(D)** Percentage of AT-MSCs showing neuron-like morphology. Representative slide showing nestin expression as determined by immunofluorescence staining **(E)** with similar expression in all groups **(F)**. Real time RT-PCR analysis showed equivalent up-regulation of neurogenic specific genes (NFM and NSE) in AT-MSCs isolated from donors of different age **(G)**. NSE: Neuron-Specific-Enolase, NFM: Neurofilament.

AT-MSC were cultured in neurogenic differentiation medium and stained with H&E (Hematoxyline and Eosin). The AT-MSCs displayed a neuronal-like differentiation with prominent and elongated neuronal structures [5] regardless of donor age (Figure 6A-C). More than 95% of the cells displayed a neuron-like morphology in each culture as indicated in Figure 6D (99.7 ± 0.3 in young vs. 95.7 ± 4.3 in adult vs. 98.0 ± 1.5 (aged) positive cells). Induction into neuron-like cells was also confirmed by the assessment of nestin expression by immunocytochemistry (Figure 6E). Again we did not observe any variation in the percentage of neuron-like positive cells between the different age groups (Figure 6F) (58.2 ± 14.4 in young vs. 53.6 ± 17.9 in adult vs. 52.4 ± 19.2 (aged) percent positive cells). A quantitative increase in mRNA levels for the neurofilament (NFM) and neuron-specific-enolase (NSE) genes [17,28] after neurogenic induction supported the observation of neuronal differentiation. However, when AT-MSCs isolated from young, adult and aged donors were compared the expression of the NFM and NSE genes were similar (Figure 6G) (NFM: 46.3 ± 16.2 N = 3 (young), 39.7 ± 6.7 (adult), 45.3 ± 17.3 (aged) fold-expression, and NSE: 24.3 ± 6.4 (young), 25.7 ± 3.5 (adult), 22.4 ± 15.2 (aged) fold-expression). Although we did not find differences between age groups when AT-MSC were induced at initial passages, other experiments indicated that younger AT-MSC cells were better than aged cells when expanded AT-MSC cultures were neurally induced at latter passages (data not shown).

## Discussion

In view of conflicting reports, we have undertaken a comprehensive analysis of age-related AT-MSC characteristics in a single study using donors of broad age range. Our study focused on the parameters of MSC yield, frequency, replication and differentiation into adipogenic, osteogenic, chondrogenic and neurogenic lineages, along with other age-related parameters (senescence, SOD activity and viability under cell stress). The present study demonstrated the effect of donor age on the cell expansion and differentiation potential of AT-MSCs, and represents one of the most comprehensive studies providing insight into whether cell-based therapies will be negatively affected by donor age. It is assumed that organismal aging is linked to diminished organ repair due to reduced functional capacity of tissue resident stem cells. It is believed that such cells residing in the elderly are subjected to age-related changes and thus contribute less to tissue rejuvenation. Similarly, age-related diseases such as diabetes and heart failure also negatively impact the function of endogenous progenitor cells [29]. As stem cells are the basis of tissue regeneration therapies, a diminished functionality of these cells in the elderly may result in reduced efficacy of autologous cell therapies. With an increase in the aging population, cellular therapies are becoming more relevant for aged patients who are the main target population for such therapies. It is therefore important to investigate donor age as a critical factor in determining whether cell therapies can achieve the desired results in these individuals using autologous stem cells.

Analysis indicated that the overall yield of nucleated cells was significantly and negatively affected by donor age. Similar observations have been reported in literature by assessing the yield of bone marrow-derived MSCs and circulating endothelial progenitor cells [30,31]. These results indicated that age-related changes in MSC number should be taken into account whenever these cells are considered for clinical applications in the elderly. Although AT-MSCs from all age groups had the ability to form colonies (an indication of cell function), AT-MSC from younger donors produced more colonies containing larger numbers of cells. Other investigators have reported that the number of cells forming colonies decreased significantly with increasing donor age and is in accordance with the results of our current study [20]. These findings are important as it indicates that harvesting AT-MSCs from elderly donors may require either the collection of more adipose tissue or require pretreatment strategies to enhance cell proliferation and expansion.

AT-MSCs isolated from each age group (young, adult and aged) exhibited a fibroblastic morphology and phenotype common to MSC that was independent of donor age [19]. That is, regardless of donor age, AT-MSC expressed CD44, CD73, CD90 and CD105 (mesenchymal markers) while lacking expression of CD3, CD14, CD19, CD34 and CD45 (hematopoietic markers). These results were in agreement with previous reports [19,20], although Stolzing et al. [24] observed age-related changes in expression of some cell surface markers such as CD44, CD90, CD105 and Stro-1 when bone marrow-derived MSCs were analyzed.

AT-MSCs obtained from aged donors displayed increased senescent features as indicated by higher expression of the p16 and p21 genes. Recent evidence suggests that p16 and p21 are markers of senescence [24]. In addition, SA-β-gal expression was also measured and was found at higher levels in aged AT-MSC cultures, while SOD activity was decreased [24]. The ability to expand cells without loss of function is one of the most important considerations when culturing MSC for therapeutic purposes. We evaluated whether donor age had an effect on proliferation. There was an age-related difference in the number of maximum population doublings, being statistically higher for AT-MSCs isolated from young donors versus adult or aged donors. AT-MSCs from young donors also proliferated at a higher rate than other groups. The doubling time of the AT-MSC was significantly increased with advanced age as well. These findings were not unexpected as the expression of other age-related markers (i.e., SA-β-gal, P16 and p21) was found to be significantly higher in aged donors compared to young donors.

Published studies have demonstrated the multi-lineage differentiation potential of AT-MSCs [5,14]. However, recent studies have raised questions about the usefulness of AT-MSCs collected from aged donors [15,20]. We thus have analyzed the differentiation capability of AT-MSCs relative to age of the donor. AT-MSCs obtained from donors of each age group efficiently differentiated into adipocytes. Quantification of oil red O content did not indicate a significant correlation between donor age and adipogenesis. The number of oil red O positive cells was also equivalent between age groups although more variation was observed in the aged group as compared to the young and adult groups. Similarly, we observed very little fluctuation in the mRNA levels of the adipogenic specific genes, PPAR-ү and LPL, due to donor age. Digirolamo et al. [32] reported similar results, as has Zhu et al. [15]. Overall, the results of our current study indicated that adipogenic potential of AT-MSCs was well preserved in advanced age and AT-MSC of any age could potentially be used for tissue engineering applications that require fat production, or potentially for cosmetic/plastic surgery applications.

Concomitant with aging, the chance of bone fractures is significantly increased while the ability to heal such fractures is lost. A significant drop in osteogenic potential of AT-MSCs with increased age was observed, as measured qualitatively by matrix calcification and quantitatively by real time RT-PCR, using osteocalcin and alkaline phosphatase gene expression. We observed a linear decrease in osteogenic potential with increasing age. Zhu et al. [15] reported a similar decline in osteogenic potential starting in middle age (40-49 years). However, Zhu et al. [15] only studied female patients with a different age range. A decrease in circulating oestrogen levels has been shown to be responsible for loss of osteogenic potential of stem cells in females [33,34]. Other conflicting reports have been published; e.g. Shi et al. [35] reported no change with age while Khan et al. [36] found age-related differences in osteogenic potential of AT-MSCs. These inconsistent results may be due to the different age ranges and the health status of the donors that were studied. Overall, the majority of reports found results similar to our current study; describing an overall decline in osteogenic potential with donor age (regardless of species).

Damaged cartilage does not heal well and stem cells might be potential candidates for cartilage repair. However, the relationship between stem cell aging and the potential to undergo chondrogenesis has not been well established. We observed that there was a significant age-related decline in the chondrogenic potential of AT-MSCs. Similarly, mRNA expression of the aggrecan and collagen type 2 genes was significantly reduced in the aged group as compared to the other groups. Murphy et al. [37] has also reported an age-related decline in chondrogenic potential of MSC similar to the results of our study. In combination, these findings and our osteogenic results indicate that donor age may negatively impact the use of AT-MSC for orthopedic applications which are not uncommon as one grows older.

Studies have indicated that MSCs could be a potential treatment for various neurodegenerative disorders. We have previously shown that hAT-MSCs can be differentiated into neuron-like cells in vitro [5]. In the current study although cell outgrowths were more prominent in young AT-MSC cultures, no significant differences were found in the total number of neuron-like cells from any age group. Similarly, we observed no significant difference in the expression of the nestin gene, as the percentage nestin expression was independent of donor age. Real time RT-PCR also indicated equivalent expression of the neuronal specific genes NFM and NSE in all age groups. Therefore, it may still be feasible to consider use of AT-MSC for neurological applications at any point in the donor’s life (e.g., for stroke, Parkinson’s disease, etc.).

## Conclusions

Stem cell research and stem cell therapy is expanding rapidly. However, a number of issues still need to be addressed to make such therapies more useful. Autologous MSC source is widely used cell source in cell based therapy for patients. However, certain limitations are applied such as poor functionality of MSCs isolated from elderly. AT-MSCs isolated from younger donors are anticipated to be a more useful cell source for tissue engineering and regenerative medicine applications. Cell based therapeutic approaches for the elderly should focus on alternative strategies such as banking younger adipose tissue for later use. Preservation of stem and progenitor cells at a younger age while when biological activity is at its greatest potential could provide an ideal situation for future regenerative medicine applications.

Overall, results of the current study indicated that aged MSCs displayed senescent features when compared with cells isolated from young donors. The results demonstrated that the growth kinetics and the osteogenic and chondrogenic potentials of AT-MSCs were adversely affected by increased donor age. However, the adipogenic and possibly the neurogenic potential of the AT-MSCs seemed to be maintained during aging.

## Abbreviations

MSCs: Mesenchymal stem cells; hAT-MSCs: Human adipose tissue derived MSCs; PDs: Population doublings; SOD: Superoxide dismutase; BM-MSCs: Bone marrow derived MSCs; PBS: Phosphate buffered saline; FBS: Fetal bovine serum; FACS: Fluorescence activated cell sorting; CFU: Colony forming unit; DT: Doubling time; PFA: Paraformaldehyde; H&E: Hematoxylin and eosin; SA-β-gal: Senescence-Associated β-galactosidase Staining; PPAR-ү: Peroxisome proliferator activated receptor-gamma; LPL: Lipoprotein lipase; NFM: Neurofilament; NSE: Neuron specific enolase.

## Competing interests

Dr. Choudhery and Ms Muise do not have financial or non-financial competing interests. Dr. Harris is the CSO for Adicyte, Inc. Dr. Badowski is a consultant to Adicyte.

## Authors’ contributions

MSC was involved in the design and experimentation of the study as was MB; AM performed experimentation and data acquisition; JP was also involved in data acquisition, experimental design and data analysis; DTH performed the overall experimental design and final data analysis. All authors have seen and agreed to the submitted version of the manuscript.

## Supplementary Material

Additional file 1: Figure S1 Phenotypic characterization. Flow cytometric analysis of cells show that AT-MSCs were positive for CD44, CD73, CD90 and CD105, while being negative for hematopoietic markers CD3, CD14, CD19, CD34 and CD45. (A) representative graphics, and (B) analysis of expression. -:<3.0%, --:<2.0%, ---:<1%, +: >93% , + +: >97.0%, + + +:>99.0%. Click here for file

Additional file 2: Table S1 The primer sequences (5′-3′) for the primer pairs used. Click here for file

## References

[B1] Pérez-Simon JA López-Villar O Andreu EJ Rifón J Muntion S Campelo MD Sánchez-Guijo FM Martinez C Valcarcel D Cañizo CD Mesenchymal stem cells expanded in vitro with human serum for the treatment of acute and chronic graft-versus-host disease: results of a phase I/II clinical trial Haematologica 2011 96 7 1072 1076 10.3324/haematol.2010.038356 21393326 PMC3128230

[B2] Choudhery MS Khan M Mahmood R Mohsin S Akhtar S Ali F Khan SN Riazuddin S Mesenchymal stem cells conditioned with glucose depletion augments their ability to repair -infarcted myocardium J Cell Mol Med 2012 16 10 2518 2529 10.1111/j.1582-4934.2012.01568.x 22435530 PMC3823444

[B3] Pittenger MF Mackay AM Beck SC Jaiswal RK Douglas R Mosca JD Moorman MA Simonetti DW Craig S Marshak DR Multilineage potential of adult human mesenchymal stem cells Science 1999 284 5411 143 147 10.1126/science.284.5411.143 10102814

[B4] Lee CC Ye F Tarantal AF Comparison of growth and differentiation of fetal and adult rhesus monkey mesenchymal stem cells Stem Cells Dev 2006 15 2 209 220 10.1089/scd.2006.15.209 16646667

[B5] Choudhery MS Badowski M Muise A Harris DT Comparison of human adipose and cord tissue derived mesenchymal stem cells Cytotherapy 2013 15 3 330 343 10.1016/j.jcyt.2012.11.010 23318344

[B6] Ryan JM Barry F Murphy JM Mahon BP Interferon-γ does not break, but promotes the immunosuppressive capacity of adult human mesenchymal stem cells Clin Exp Immunol 2007 149 2 353 363 10.1111/j.1365-2249.2007.03422.x 17521318 PMC1941956

[B7] Abumaree M Al Jumah M Pace RA Kalionis B Immunosuppressive properties of mesenchymal stem cells Stem Cell Rev 2012 8 2 375 392 10.1007/s12015-011-9312-0 21892603

[B8] Amado LC Saliaris AP Schuleri KH St John M Xie JS Cattaneo S Durand DJ Fitton T Kuang JQ Stewart G Lehrke S Baumgartner WW Martin BJ Heldman AW Hare JM Cardiac repair with intramyocardial injection of allogeneic mesenchymal stem cells after myocardial infarction Proc Natl Acad Sci U S A 2005 102 32 11474 11479 10.1073/pnas.0504388102 16061805 PMC1183573

[B9] Xin H Li Y Shen LH Liu X Wang X Zhang J Pourabdollah-Nejad DS Zhang C Zhang L Jiang H Zhang ZG Chopp M Increasing tPA activity in astrocytes induced by multipotent mesenchymal stromal cells facilitate neurite outgrowth after stroke in the mouse PLoS One 2010 5 2 e9027 10.1371/journal.pone.0009027 20140248 PMC2815778

[B10] Taylor SE Smith RK Clegg PD Mesenchymal stem cell therapy in equine musculoskeletal disease: scientific fact or clinical fiction? Equine Vet J 2007 39 2 172 180 10.2746/042516407X180868 17378447

[B11] Kretlow JD Jin YQ Liu W Zhang WJ Hong TH Zhou G Baggett LS Mikos AG Cao Y Donor age and cell passage affects differentiation potential of murine bone marrow-derived stem cells BMC Cell Biol 2008 9 60 10.1186/1471-2121-9-60 18957087 PMC2584028

[B12] Choudhery MS Khan M Mahmood R Mehmood A Khan SN Riazuddin S Bone marrow derived mesenchymal stem cells from aged mice have reduced wound healing, angiogenesis, proliferation and anti-apoptosis capabilities Cell Biol Int 2012 36 8 747 753 10.1042/CBI20110183 22352320

[B13] Zuk PA Zhu M Ashjian P De Ugarte DA Huang JI Mizuno H Alfonso ZC Fraser JK Benhaim P Hedrick MH Human adipose tissue is a source of multipotent stem cells Mol Biol Cell 2002 13 12 4279 4295 10.1091/mbc.E02-02-0105 12475952 PMC138633

[B14] Zuk PA Zhu M Mizuno H Huang J Futrell JW Katz AJ Benhaim P Lorenz HP Hedrick MH Multi-lineage cells from human adipose tissue: implications for cell-based therapies Tissue Eng 2001 7 2 211 228 10.1089/107632701300062859 11304456

[B15] Zhu M Kohan E Bradley J Hedrick M Benhaim P Zuk P The effect of age on osteogenic, adipogenic and proliferative potential of female adipose-derived stem cells J Tissue Eng Regen Med 2009 3 4 290 301 10.1002/term.165 19309766

[B16] Giovannini S Diaz-Romero J Aigner T Heini P Mainil-Varlet P Nesic D Micromass co-culture of human articular chondrocytes and human bone marrow mesenchymal stem cells to investigate stable neocartilage tissue formation in vitro Eur Cell Mater 2010 20 245 259 20925023 10.22203/ecm.v020a20

[B17] Kim WK Jung H Kim DH Kim EY Chung JW Cho YS Park SG Park BC Ko Y Bae KH Lee SC Regulation of adipogenic differentiation by LAR tyrosine phosphatase in human mesenchymal stem cells and 3T3-L1 preadipoctes J Cell Sci 2009 122 pt 22 4160 4167 19910497 10.1242/jcs.053009

[B18] Nalesso G Sherwood J Bertrand J Pap T Ramachandran M De Bari C Pitzalis C Dell’accio F WNT-3A modulates articular chondrocyte phenotype by activating both canonical and noncanonical pathways J Cell Biol 2011 193 3 551 564 10.1083/jcb.201011051 21536751 PMC3087013

[B19] Dominici M Le Blanc K Mueller I Slaper-Cortenbach I Marini F Krause D Deans R Keating A Prockop DJ Horwitz E Minimal criteria for defining multipotent mesenchymal stromal cells: the international society for cellular therapy position statement Cytotherapy 2006 8 4 315 317 10.1080/14653240600855905 16923606

[B20] Alt EU Senst C Murthy SN Slakey DP Dupin CL Chaffin AE Kadowitz PJ Izadpanah R Aging alters tissue resident mesenchymal stem cell properties Stem Cell Res 2012 8 2 215 225 10.1016/j.scr.2011.11.002 22265741

[B21] Capparelli C Chiavarina B Whitaker-Menezes D Pestell TG Pestell RG Hulit J Andò S Howell A Martinez-Outschoorn UE Sotgia F Lisanti MP CDK inhibitors (p16/p19/p21) induce senescence and autophagy in cancer-associated fibroblasts, “fueling” tumor growth via paracrine interactions, without an increase in neo-angiogenesis Cell Cycle 2012 11 19 3599 3610 10.4161/cc.21884 22935696 PMC3478311

[B22] Dimri GP Lee X Basile G Acosta M Scott G Roskelley C Medrano EE Linskens M Rubelj I Pereira-Smith O A biomarker that identifies senescent human cells in culture and in aging skin in vivo Proc Natl Acad Sci U S A 1995 92 20 9363 9367 10.1073/pnas.92.20.9363 7568133 PMC40985

[B23] Menicanin D Bartold PM Zannettino AC Gronthos S Genomic profiling of mesenchymal stem cells Stem Cell Rev Rep 2009 5 1 36 50 10.1007/s12015-009-9056-2 19224407

[B24] Stolzing A Jones E McGonagle D Scutt A Age-related changes in human bone marrow derived mesenchymal stem cells: consequences for cell therapies Mech Ageing Dev 2008 129 3 163 173 10.1016/j.mad.2007.12.002 18241911

[B25] Jurgens WJ Oedayrajsingh-Varma MJ Helder MN Zandiehdoulabi B Schouten TE Kuik DJ Ritt MJ Van Milligen FJ Effect of tissue-harvesting site on yield of stem cells derived from adipose tissue: implications for cell-based therapies Cell Tissue Res 2008 332 3 415 426 10.1007/s00441-007-0555-7 18379826 PMC2386754

[B26] Gonzalez R Griparic L Vargas V Burgee K Santacruz P Anderson R Schiewe M Silva F Patel A A putative mesenchymal stem cells population isolated from adult human testes Biochem Biophys Res Commun 2009 385 4 570 575 10.1016/j.bbrc.2009.05.103 19482010

[B27] Erickson GR Gimble JM Franklin DM Rice HE Awad H Guilak F Chondrogenic potential of adipose tissue-derived stromal cells in vitro and in vivo Biochem Biophys Res Commun 2002 290 2 763 769 10.1006/bbrc.2001.6270 11785965

[B28] Jang S Cho HH Cho YB Park JS Jeong HS Functional neural differentiation of human adipose tissue-derived stem cells using bFGF and forskolin BMC Cell Biol 2010 11 25 10.1186/1471-2121-11-25 20398362 PMC2867791

[B29] Dimmeler S Leri A Aging and disease as modifiers of efficacy of cell therapy Circ Res 2008 102 11 1319 1330 10.1161/CIRCRESAHA.108.175943 18535269 PMC2728476

[B30] Scheubel RJ Zorn H Silber RE Kuss O Morawietz H Holtz J Simm A Age-dependent depression in circulating endothelial progenitor cells in patients undergoing coronary artery bypass grafting J Am Coll Cardiol 2003 42 12 2073 2080 10.1016/j.jacc.2003.07.025 14680729

[B31] Tokalov SV Grüner S Schindler S Wolf G Baumann M Abolmaali N Age-related changes in the frequency of mesenchymal stem cells in the bone marrow of rats Stem Cells Dev 2003 16 3 439 446 17610374 10.1089/scd.2006.0078

[B32] Digirolamo CM Stokes D Colter D Phinney DG Class R Prockop DJ Propagation and senescence of human marrow stromal cells in culture: a simple colony-forming assay identifies samples with the greatest potential to propagate and differentiate Br J Haematol 1999 107 2 275 281 10.1046/j.1365-2141.1999.01715.x 10583212

[B33] Robinson JA Harris SA Riggs BL Spelsberg TC Estrogen regulation of human osteoblastic cell proliferation and differentiation Endocrinology 1997 138 7 2919 2917 9202236 10.1210/endo.138.7.5277

[B34] Ankrom MA Patterson JA d’Avis PY Vetter UK Blackman MR Sponseller PD Tayback M Robey PG Shapiro JR Fedarko NS Age-related changes in human oestrogen receptor α function and levels in steoblasts Biochem J 1998 333 Pt 3 787 794 9677341 10.1042/bj3330787 PMC1219645

[B35] Shi YY Nacamuli RP Salim A Longaker MT The osteogenic potential of adipose-derived mesenchymal cells is maintained with aging Plast Reconstr Surg 2005 116 6 1686 1696 10.1097/01.prs.0000185606.03222.a9 16267433

[B36] Khan WS Adesida AB Tew SR Andrew JG Hardingham TE The epitope characterisation and the osteogenic differentiation potential of human fat pad-derived stem cells is maintained with ageing in later life Injury 2009 40 2 150 157 10.1016/j.injury.2008.05.029 19070850

[B37] Murphy JM Dixon K Beck S Fabian D Feldman A Barry F Reduced chondrogenic and adipogenic activity of mesenchymal stem cells from patients with advanced osteoarthritis Arthritis Rheum 2002 46 3 704 713 10.1002/art.10118 11920406

