# Supplementary figures and images for: Donor age negatively impacts adipose tissue-derived mesenchymal stem cell expansion and differentiation
Source: J Transl Med. 2014 Jan 7;12:8. doi: 10.1186/1479-5876-12-8 (PMC3895760; doi:10.1186/1479-5876-12-8)

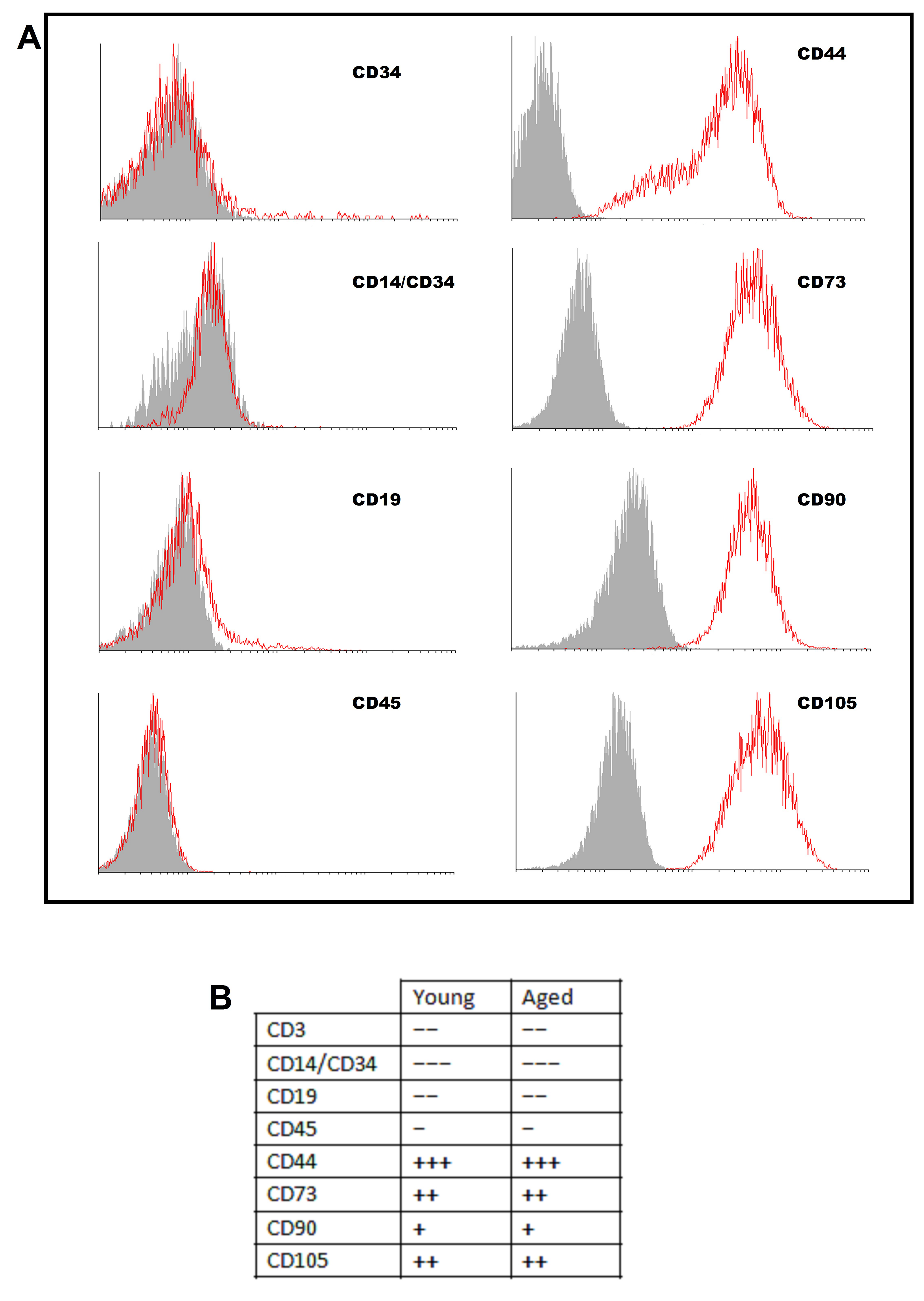

Supplement: Additional file 1: Figure S1 — Phenotypic characterization. Flow cytometric analysis of cells show that AT-MSCs were positive for CD44, CD73, CD90 and CD105, while being negative for hematopoietic markers CD3, CD14, CD19, CD34 and CD45. (A) representative graphics, and (B) analysis of expression. -:<3.0%, --:<2.0%, ---:<1%, +: >93% , + +: >97.0%, + + +:>99.0%. [file 1479-5876-12-8-S1.tiff]
