# Supplementary material for: Donor age negatively impacts adipose tissue-derived mesenchymal stem cell expansion and differentiation
Source: J Transl Med. 2014 Jan 7;12:8. doi: 10.1186/1479-5876-12-8 (PMC3895760; doi:10.1186/1479-5876-12-8)
Supplement: Additional file 2: Table S1 — The primer sequences (5′-3′) for the primer pairs used. [file 1479-5876-12-8-S2.doc]

| No. | Gene | Primer Sequences |
| --- | --- | --- |
| 1 | Coll 2 | GGCAATAGCAGGTTCACGTACA (F) |
|  |  | CGATAACAGTCTTGCCCCACTT (R) |
| 2 | PPAR-γ | AAGACCACTCCCACTCCTTTG (F) |
|  |  | GTCAGCGGACTCTGGATTCA (R) |
| 3 | LPL | GTCCGTGGCTACCTGTCATT (F) |
|  |  | TGTCCCACCAGTTTGGTGTA (R) |
| 4 | Osteocalcin | GGCAGCGAGGTAGTGAAGAG (F) |
|  |  | CTGGAGAGGAGCAGAACTGG (R) |
| 5 | Aggrecan | TCAACAACAATGCCCAAGAC (F) |
|  |  | AGCGACAAGAAGAGGACACC (R) |
| 6 | Alkaline Phosphatase | GACCCTTGACCCCCACAAT (F) |
|  |  | GCTCGTACTGCATGTCCCCT (R) |
| 7 | Beta Actin | AGAGCTACGAGCTGCCTGAC (F) |
|  |  | AGTACTTGCGCTCAGGAGGA (R) |
